# Reactive Oxygen Species in Pathogen Clearance: The Killing Mechanisms, the Adaption Response, and the Side Effects

**DOI:** 10.3389/fmicb.2020.622534

**Published:** 2021-02-04

**Authors:** Hao Li, Xuedong Zhou, Yuyao Huang, Binyou Liao, Lei Cheng, Biao Ren

**Affiliations:** State Key Laboratory of Oral Diseases, National Clinical Research Center for Oral Diseases, West China Hospital of Stomatology, Sichuan University, Chengdu, China

**Keywords:** reactive oxygen species, secondary damage, metabolism remodeling, virulence, antibiotic resistance, antibiotic tolerance

## Abstract

Reactive oxygen species (ROS) are attractive weapons in both antibiotic-mediated killing and host-mediated killing. However, the involvement of ROS in antibiotic-mediated killing and complexities in host environments challenge the paradigm. In the case of bacterial pathogens, the examples of some certain pathogens thriving under ROS conditions prompt us to focus on the adaption mechanism that pathogens evolve to cope with ROS. Based on these, we here summarized the mechanisms of ROS-mediated killing of either antibiotics or the host, the examples of bacterial adaption that successful pathogens evolved to defend or thrive under ROS conditions, and the potential side effects of ROS in pathogen clearance. A brief section for new antibacterial strategies centered around ROS was also addressed.

## Introduction

Reactive oxygen species (ROS), including hydrogen peroxide (H_2_O_2_), hydroxyl radical (OH-), singlet oxygen (^1^O_2_), and superoxide anion (⋅O_2_^–^), are produced by the pathogen itself (endogenous) as a byproduct of aerobic respiration; they can also be encountered in the host environment (exogenous). ROS have been called “double-edged swords of life” (Mittler, 2017) in pathogen clearance. First, since ROS can directly damage DNA, lipids, and proteins, they are thought to be the weapon used by both antibiotics and the host immune system. However, controversies challenge the paradigm. Second, successful pathogens exploit ROS for their own adaption. This minireview aims to summarize the mechanisms of ROS-mediated killing by either antibiotics or the host, examples of pathogens that cope with the ROS conditions, the potential side effects of ROS in pathogen clearance, and novel antibacterial strategies centered around ROS.

## Endogenous ROS, As Secondary Damage, Formed in Response to Antibiotic Exposure

In general, antibiotics are thought to kill microbes through interaction with specific intracellular targets (Kohanski et al., 2010b). However, in 2007, Collins group proposed a novel mechanism of quinolones-induced killing by the generation of endogenous hydroxyl radicals (Dwyer et al., 2007). Since then, ROS have been shown to play a central role in lethality of numerous classes of bactericidal antibiotics, regardless of their specific targets (Kohanski et al., 2007). However, the role of ROS in antibiotic lethality became controversial and has been challenged (Keren et al., 2013; Imlay, 2015), since antibiotics can kill in the absence of ROS. The paradox contained in these statements can be solved by the idea that killing can derive from the primary damage of antibiotic or from a secondary, lethal stress response mediated by ROS (Zhao and Drlica, 2014; Zhao et al., 2015; Luan et al., 2018). If primary damage with specific targets is severe enough, it can result in death directly. Otherwise, primary damage stimulates a pathway that leads to ROS accumulation as secondary damage. New evidence for this hypothesis was provided in *Escherichia coli*. Hong et al. (2019) described a novel experimental system in which the role of secondary ROS damage could be tested in isolation. In this case, even after complete removal of quinolones, ROS accumulation and cell death continued to occur, indicating that when secondary ROS damage exceeds a critical threshold, it becomes a self-amplifying process and the terminal stage when bacteria responds to antibiotics.

However, how primary damage leads to ROS accumulation remained unclear. Since target-specific damage occurs in antibiotic killing, pathways leading to ROS accumulation most probably have drug-specific context as well. While different studies suggest that primary drug-target damage may activate processes such as envelopes stress response and programmed cell death, leading to ROS accumulation (Lobritz et al., 2015; Van Acker and Coenye, 2017; Stokes et al., 2019), most evidence is provided for aminoglycosides. Misfolded proteins induced by gentamicin first insert into the membrane in *E. coli* and lead the activation of the response regulator ArcA through CpxA, the envelope stress response sensor. ArcA then activates the tricarboxylic acid (TCA) cycle enzymes, leading to dysfunction of TCA cycle and the hyperactivation of respiration (Kohanski et al., 2008). Similar dysfunction of TCA cycle can also be observed in quinolones and β-lactams treatments, suggesting that such metabolic flux could be a shared mechanism pushing the cell into a state that provokes oxidative stress in antibiotic treatments (Kohanski et al., 2008; Belenky et al., 2015). While β-lactams may directly activate envelope stress response by affecting membrane integrity, specific triggers for quinolones remain to be worked out. Several studies found that programmed cell death mediated by YihE kinase and MazF toxin was linked to a ROS cascade in quinolone treatment (Drlica et al., 2008; Dorsey-Oresto et al., 2013). Recently, additional evidence suggested that quinolones disrupted the nucleotide pool, leading to the increase of ATP demand (Yang et al., 2019). The increasing ATP demand elevates TCA cycle activity and cellular respiration and enhances antibiotic lethality.

## Exogenous ROS-Induced Killing Depends on a Variety of Innate Mechanisms

Exogenous ROS, as an antimicrobial weapon wielded by phagocytes, are generated from NADPH oxidase (NOX2) in response to microbe recognition (Panday et al., 2015). However, it has been surprisingly difficult to figure out exactly how phagocytic ROS production suppresses microbial growth (Imlay, 2019). Some indirect evidence support the notion that phagocytic ROS directly kill pathogens. Chronic granulomatous disease (CGD) is a genetic disorder in which patients lack functional NOX2 protein and therefore are associated with impaired respiratory burst (Nguyen et al., 2017). Indeed, a severe neutrophil killing defect was reported in CGD patients (Dinauer, 2005; Klebanoff, 2005). The host susceptibility to various pathogens including *Salmonella enterica*, *Staphylococcus aureus*, and *Burkholderia cepacia* in CGD patients highlights the importance of the respiratory burst to infectious diseases. However, whether host-derived ROS could directly kill pathogens is still a matter of debate, since several studies argued that intracellular ROS level in phagosomes was insufficient to kill pathogens (Slauch, 2011; Li and Imlay, 2018; Imlay, 2019).

Alternatively, ROS generated by NOX, as a signal, promote pathogen elimination via activating a variety of innate and adaptive mechanisms in the host cell. For example, ROS are commonly believed to be required in autophagy. NOX-derived ROS are indispensable for the recruitment of the autophagy protein light chain 3 (LC3), promoting antibacterial autophagy (Huang et al., 2009; de Luca et al., 2014). In addition, during infection, neutrophils and phagocytes can activate an additional antimicrobial mechanism, referred to as neutrophil extracellular traps (NETs) (Stoiber et al., 2015). NETs, formed by chromatin and associated proteins, trap and kill various extracellular pathogens. NET formation requires the production of ROS (Papayannopoulos et al., 2010) and is impaired in NOX2-deficient neutrophils (Stoiber et al., 2015). Another study suggested that fosfomycin, a broad-spectrum antibacterial agent, could enhance NET-mediated killing of *S. aureus* via NOX2-dependent ROS accumulation in mouse model (Shen et al., 2016).

In addition to the NOX complex, the mitochondrion is another cellular source of ROS in immune cells (Abuaita et al., 2018). Upon macrophage activation, mitochondrial conditions favor reverse electron transport in the electron transport chain and thus generate ROS (Garaude et al., 2016; Pinegin et al., 2018). Mice deficient in proteins responsible for generation of mitoROS are highly susceptible to infections caused by *Salmonella typhimurium* and *Listeria monocytogenes* (Sonoda et al., 2007; West et al., 2011). During methicillin-resistant *S. aureus* (MRSA) infection, mitoROS were generated and delivered directly to the phagosome by mitochondrial-derived vesicles (MDVs) in a Toll-like receptor signaling-dependent manner (Abuaita et al., 2018). Of note, like NOX-mediated ROS, which can modulate IL-1β expression (Warnatsch et al., 2017), mitoROS can also modulate the antimicrobial functions of innate immune cells by regulating production of multiple cytokines both *in vitro* and *in vivo* (Mills et al., 2016; Herb et al., 2019).

## Bacterial Response to Oxidative Stress: Antioxidant and Metabolic Defenses

The systems in bacteria that regulate the expression of antioxidant defense networks have been extensively reviewed (Ezraty et al., 2017; Reniere, 2018). The ROS response is under the control of the master regulators. Transcription factors such as OxyR (i.e., in *S. enterica*, *Francisella tularensis*, and *Porphyromonas gingivalis*), PerR (i.e., in *S. aureus* and *Bacillus subtilis*), OhrR (i.e., in *B. subtilis* and *Mycobacterium smegmatis*), and SoxRS (i.e., in *E. coli* and *S. aureus*) can be activated by direct oxidation of their sensor proteins and then adjusting the bacterial response appropriately (Chiang and Schellhorn, 2012). Although the particulars vary among different species, in general, these regulons regulate genes required for antioxidant defense, including superoxide dismutase, catalase, thioredoxins, heme biosynthesis machinery, glutathione reductases, ferric uptake regulator (Fur), ferritin, and bacterioferritin (Imlay, 2008; Chiang and Schellhorn, 2012; Reniere, 2018). In addition, iron homeostasis is also critical to mitigate redox damage induced by Fenton reaction. Therefore, in pathogenic bacteria (i.e., *E. coli*, *S. aureus*, and *Salmonella*), the iron-sensing transcriptional repressors, such as Fur and DtxR (diphtheria toxin repressor), can also be utilized to sustain redox homeostasis by controlling the expression of genes encoding iron acquisition systems and iron-dependent enzymes (Troxell and Hassan, 2013).

**FIGURE 1:**
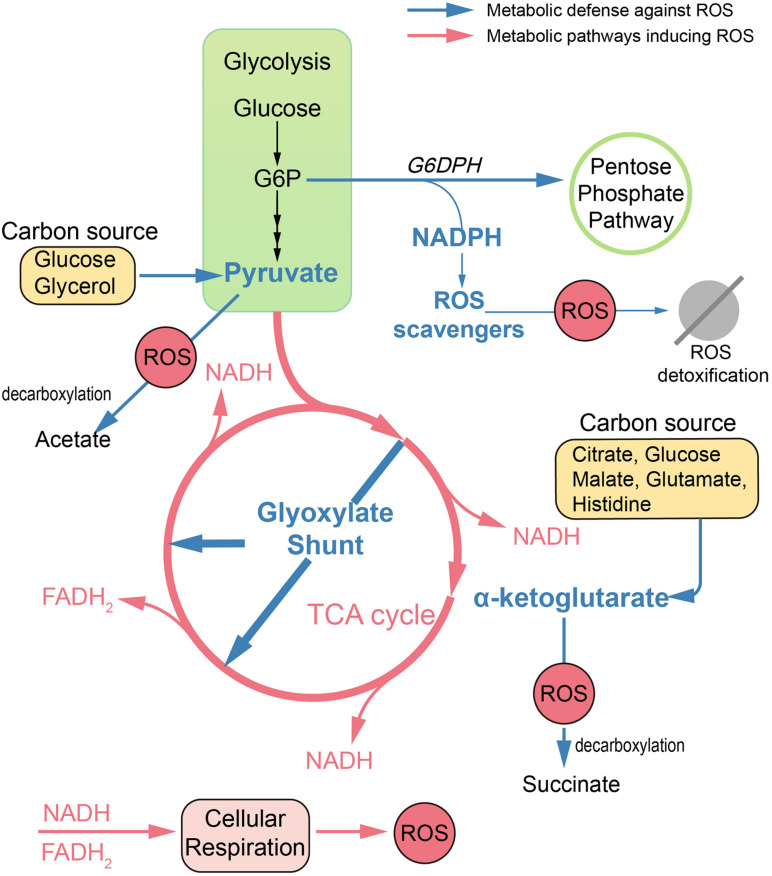
Metabolic remodeling designed to reduce oxidative damage. In antibiotic treatments, dysfunction of TCA cycle and hyperactivation of cell respiration lead to ROS accumulation in bacteria. Defense against ROS can be achieved by metabolic remodeling. Reduced endogenous ROS formation can be achieved by upregulation of the glyoxylate shunt, which bypasses two NADH-generating steps. Replenishment of antioxidants can be achieved by rerouting metabolism toward the pentose phosphate pathway and enhancing production of cofactor NADPH. Also, ketoacids, including pyruvate and α-ketoglutarate, can mitigate oxidative damage via non-enzymatic decarboxylation in the presence of numerous carbon sources. G6P, glucose 6-phosphate; G6DPH, glucose-6-phosphate dehydrogenase.

Apart from antioxidant defense systems, metabolism remodeling also plays a pivotal role in mitigating oxidative damage (Figure 1). Metabolic adaptions can reduce oxidative burden by retarding respiration. For example, the glyoxylate shunt (GS) is an anaplerotic reaction of the TCA cycle developed in numerous species, which bypasses two NADH-generating steps (Lemire et al., 2017; Dolan and Welch, 2018). In *B. cepacia*, GS genes were upregulated in cells surviving aminoglycoside treatment (Van Acker et al., 2013). Similarly, in *Mycobacterium tuberculosis* (MTB), GS enzyme isocitrate lyase deficient mutants were significantly more susceptible than wild-type strain toward isoniazid, rifampicin, and streptomycin (Nandakumar et al., 2014). Consistent with these observations, the Collins group found that glyoxylate could serve as a direct biochemical inducer of aminoglycoside tolerance via reducing cellular respiration. Meanwhile, TCA cycle intermediates, such as fumarate, significantly potentiate tobramycin lethality (Meylan et al., 2017).

Replenishment of antioxidants can also be achieved by metabolic modulation. Pentose phosphate (PP) pathway is an important target to mitigate ROS damage, since NADPH is the cofactor for antioxidant systems. In *E. coli*, by increased abundance of glucose-6-phosphate dehydrogenase, the metabolic flux can be rerouted toward the PP pathway, leading to increased ROS tolerance (Christodoulou et al., 2018).

Ketoacids including α-ketoglutarate (KG) and pyruvate can undergo non-enzymatic decarboxylation in the presence of ROS, generate non-toxic byproducts, and thus alleviate the ROS damage. Bacteria like *E. coli* and *Pseudomonas fluorescens* tend to pool ketoacids both inside the cell and in the extracellular matrix under ROS conditions. For example, the increased generation of KG can be achieved by the modulation of TCA cycle enzymes like isocitrate dehydrogenase when using citrate, glucose, and malate as carbon sources (Alhasawi et al., 2016). It can also be achieved by the deamination of glutamate when glutamate or histidine is supplied as the carbon source (Lemire et al., 2010, 2017). When exposed to H_2_O_2_, *P. fluorescens* can go through a metabolic reconfiguration with enhanced activity of substrate-level phosphorylation as well as impaired activity of the TCA cycle, leading to evident pyruvate synthesis with glucose or glycerol as the carbon source (Bignucolo et al., 2013; Alhasawi et al., 2016).

## Induction of Bacterial Virulence Under ROS Conditions

In addition to antioxidant defenses, activations of the oxidative defense regulators are also required for full virulence in pathogens. For example, OxyR contributes to the virulence of *E. coli* and *Pseudomonas aeruginosa* (Lau et al., 2005; Wei et al., 2012; Fang et al., 2016). Similarly, SoxRS has been shown to act as a positive regulator in *Salmonella* pathogenicity island (SPI)-2 mediated virulence in *S. enterica* (Wang P. et al., 2020). In *S. aureus*, after oxidation of the sensor protein by ROS, the redox-signaling regulator AirSR positively regulates the biosynthesis of staphyloxanthin (STX), an important virulence factor of *S. aureus* (Hall et al., 2017). MntR, a member of the DtxR family, is required for *S. aureus* pathogenesis via maintaining manganese homeostasis (Grunenwald et al., 2019).

In host conditions, virulence modulation can be achieved by ROS indirectly. During ROS generation, phagocytes simultaneously produce glutathione (GSH) to sustain intracellular redox homeostasis. Certain intracellular pathogens have even evolved to exploit the host GSH for adaptions. In *Burkholderia pseudomallei*, virulence is completely dependent on the expression of a type VI secretion system (T6SS). During the exit from phagosome, *B*. *pseudomallei* senses host GSH via sensor protein VirA, which then activates the expression of the T6SS (Wong et al., 2015). In *L. monocytogenes*, the extracellular host GSH can trigger the production of bacterial GSH. Both host GSH and bacterial GSH bind to the master virulence regulator PrfA and function as an allosteric activator (Reniere et al., 2015; Portman et al., 2017).

## Thrive Under ROS Conditions by Metabolic Remodeling

While pathogens can be eliminated by ROS, it is quite clear that certain pathogens exploit ROS to coordinate metabolism to thrive. For example, *S. typhimurium* evidently makes use of host-derived ROS during intestinal inflammation. ROS generated by phagocytes convert thiosulfate to tetrathionate, which in turn can be used as a respiratory electron sink by *S. typhimurium*, allowing it to outcompete the native microbiota (Winter et al., 2010; Bäumler and Sperandio, 2016). Another example comes from *E. coli*. In the intestinal lumen, oxygen from the epithelium collides with sulfide generated by luminal bacteria, potentially generating H_2_O_2_ through direct reaction. Also, lactic-acid bacteria excrete H_2_O_2_ as a direct metabolic product. Under such circumstances, cytochrome *c* peroxidase (Ccp) regulated by OxyR allows *E. coli* to employ H_2_O_2_ as a terminal oxidant for respiration (Khademian and Imlay, 2017). *Helicobacter pylori* is well known as a ROS-inducing gastric pathogen. It utilizes chemotaxis to seek sites optimal for efficient colonization. Recent studies showed that ROS could be sensed in *H. pylori* by the chemoreceptor TlpD. Host oxidants hypochlorous acid (HOCl) could act as a chemoattractant by reversibly oxidizing TlpD that inactivates the chemotransduction signaling complex (Perkins et al., 2019). While H_2_O_2_ could act as a chemorepellent which initiates chemotaxis through TlpD to promote gastric gland colonization (Collins et al., 2018).

## Bacterial Resistance and Tolerance as Side Effects of ROS

Recently, the radical-based theory in pathogen clearance has been questioned for the protective role of ROS against antimicrobial killing (Burger and Drlica, 2009; Mosel et al., 2013). ROS can directly damage DNA, leading to genetic mutations. Indeed, ROS produced by non-lethal antibiotics induce mutations in specific antibiotic targets and promote the development of multi-drug resistance (Kohanski et al., 2010a; Takahashi et al., 2017). If ROS is a shared mechanism in antibiotic lethality, then it is to be expected that protection against ROS can be one of the shared traits for bacterial resistance against antibiotics as well. Indeed, bactericidal antibiotic mediates *de novo* acquisition of resistance, which then provides protection against ROS accumulation upon exposure to a different type of antibiotics (Hoeksema et al., 2018). In addition, it was recently demonstrated that non-lethal exposure to H_2_O_2_ boosted evolvability of bacterial populations by enhancing survival under oxidative stress (Rodriguez-Rojas et al., 2020).

ROS also act as a signal for gene transformation. H_2_O_2_ is a metabolic product of certain oral streptococci such as *Streptococcus gordonii* under aerobic conditions. In H_2_O_2_-producing streptococci, the release of extracellular DNA (eDNA) is entirely dependent on H_2_O_2_. Also, numerous H_2_O_2_-non-producing species like *Streptococcus mutans* and *Streptococcus pyogenes* release eDNA in a H_2_O_2_-dependent manner (Itzek et al., 2011), suggesting that ROS serve as an important environmental signal in oral biofilm (Redanz et al., 2018). Intriguingly, a recent study shows that non-antibiotic pharmaceuticals including non-steroidal anti-inflammatory drugs (NSAIDs) and the lipid-lowering drug also induce significant ROS accumulation in *Acinetobacter baylyi*, which is closely related to the enhanced transformation of antibiotic resistance genes (ARGs) (Wang Y. et al., 2020).

ROS are key weapons in host cells, while recent evidence suggests that host ROS induce antibiotic tolerance during infection. ROS generated by macrophages target TCA enzymes aconitase and succinate dehydrogenase and coerce *S. aureus* into a metabolic state with reduced respiration, which is incompatible with the killing mechanism of most bactericidal antibiotics (Rowe et al., 2019). The persister is an extreme case of tolerance for the high levels of multi-drug tolerance (Brauner et al., 2016). Host ROS can also modulate persister formation in *E. coli*. The activation of redox regulators SoxRS results in increasing expression of the AcrAB-TolC multidrug-resistant (MDR) pump, which in turn lowers the fluoroquinolones concentration and promotes antibiotic tolerance (Wu et al., 2012).

**FIGURE 2:**
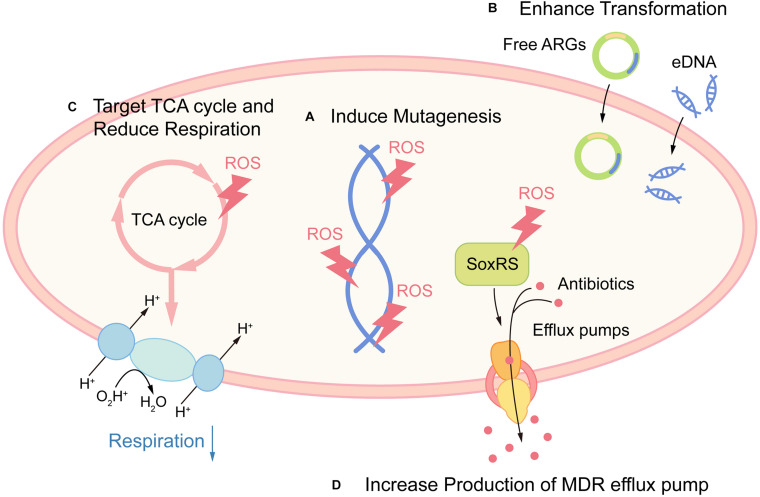
Promoting antibiotic resistance and tolerance as potential side effects of ROS. **(A)** ROS promote resistance by directly damaging DNA and leading to mutagenesis. **(B)** ROS act as a signal for gene transformation, by promoting the transformation of antibiotic resistance genes (ARGs) and eDNA in certain species. **(C)** Host-derived ROS promote antibiotic tolerance by targeting TCA cycle and retarding cellular respiration in *S. aureus*. **(D)** Host-derived ROS promote antibiotic tolerance by increased production of MDR efflux pump via activation of redox regulon SoxRS.

Together, these examples indicate that ROS may potentiate the emergence of bacterial resistance and tolerance (Figure 2). Although there is currently no clinical evidence proving this hypothesis, contradictory evidence favoring these potential side effects have been indicated in several studies. Early study suggested that *S. aureus* internalized in leucocytes isolated from patients with CGD (with impaired respiratory burst) were more susceptible to rifampicin, when compared with wild-type leucocytes (Jacobs and Wilson, 1983). Also, elevated levels of oxidation products are related to MTB infection in pulmonary tuberculosis (PTB) patients (Amaral et al., 2016). While there is scarce demonstration that the use of antioxidants can increase pathogen burden, a study showed that the antioxidant resveratrol reduced *Serratia marcescens* burden in mice (Lu et al., 2008). Surprisingly, the use of antioxidant N-acetyl cysteine (NAC) as an adjuvant to directly observed treatment short course significantly caused early sputum negativity in PTB patients (Mahakalkar et al., 2017).

## Novel Antimicrobial Strategies Centered Around ROS

The efficacy of antibiotics has been endangered by the rapid emergence of resistant pathogens. Therefore, new approaches to fight against pathogens, including antimicrobial photodynamic therapy (aPDT) and cold atmospheric plasma (CAP), have been suggested as efficient alternative approaches. Although their antibacterial efficacy also centered around ROS, the modes of action for these new techniques are distinct from conventional antibiotics, as ROS-mediated damage in such therapies is the primary stress-induced damage (Wilson and Patterson, 2008; Vatansever et al., 2013). For example, aPDT uses photosensitizers (PS) to generate ROS upon irradiation by visible light at specific wavelength, and CAPs are partly ionized gases producing a reactive mix by interacting with oxygen, generating a cocktail of ROS (Brany et al., 2020). Due to direct redox-active properties, aPDT and CAP can cause multi-target oxidative damage to pathogens (Melo et al., 2013; Hu et al., 2018) as well as direct oxidation of polysaccharides in biofilm (Beirão et al., 2014; Jiao et al., 2019). Due to their multi-target mode of action and large quantities of ROS production which overwhelm the antioxidant defenses in pathogens, the additional advantage of these new techniques is the lack of development of resistance mechanisms (Tavares et al., 2010; Kvam et al., 2012). As novel ROS-inducing strategies, aPDT and CAP showed excellent potential to tackle difficult-to-eradicate infections as alternative treatments in wound healing, dental cure, and food decontamination (Wilson and Patterson, 2008; Rao et al., 2020).

## Concluding Remarks

ROS are attractive weapons to kill pathogenic microbial cells. However, ROS, as a double-edged sword, should be regulated with care since under non-lethal ROS, certain pathogens have evolved delicate mechanisms to utilize ROS for their adaption to thrive. Also, numerous pathogens can use ROS as a stepstone for the evolution of antibiotic tolerance and resistance. Efforts to target bacterial adaptive pathways, as well as the use of novel ROS-inducing antibacterial strategies, will be promising approaches for antibacterial therapy.

## Author Contributions

HL, XZ, YH, BL, LC, and BR: conception/design of the work, agreement to be accountable for all aspects of the work. HL, LC, and BR: drafting the work. XZ, LC, and BR: final approval of the manuscript to be published. All authors contributed to the article and approved the submitted version.

## Conflict of Interest

The authors declare that the research was conducted in the absence of any commercial or financial relationships that could be construed as a potential conflict of interest.
