# Corrigendum: Reactive Oxygen Species in Pathogen Clearance: The Killing Mechanisms, the Adaption Response, and the Side Effects

**DOI:** 10.3389/fmicb.2021.685133

**Published:** 2021-05-13

**Authors:** Hao Li, Xuedong Zhou, Yuyao Huang, Binyou Liao, Lei Cheng, Biao Ren

**Affiliations:** State Key Laboratory of Oral Diseases, National Clinical Research Center for Oral Diseases, West China Hospital of Stomatology, Sichuan University, Chengdu, China

**Keywords:** reactive oxygen species, secondary damage, metabolism remodeling, virulence, antibiotic resistance, antibiotic tolerance

In the original article, there was an error. The original article read: Recent study shows that host ROS can be sensed as a chemorepellent in *H. pylori* by the chemoreceptor TlpD, which initiates chemotaxis to promote gastric gland colonization (Collins et al., 2018; Perkins et al., 2019).

A correction has been made to the section, ***Thrive Under ROS Conditions by Metabolic*** ***Remodeling*. **The corrected sentence is below:

Recent studies showed that ROS could be sensed in *H. pylori* by the chemoreceptor TlpD. Host oxidants hypochlorous acid (HOCl) could act as a chemoattractant by reversibly oxidizing TlpD that inactivates the chemotransduction signaling complex (Perkins et al., 2019). While H_2_O_2_ could act as a chemorepellent which initiates chemotaxis through TlpD to promote gastric gland colonization (Collins et al., 2018).

The authors apologize for this error and state that this does not change the scientific conclusions of the article in any way. The original article has been updated.
